# Estimating metabolite networks subject to dietary preferences and lifestyle

**DOI:** 10.1007/s11306-025-02296-2

**Published:** 2025-08-11

**Authors:** Georgios Bartzis, Carel F. W. Peeters, Hae-Won Uh, Jeanine J. Houwing-Duistermaat, Fred A. van Eeuwijk

**Affiliations:** 1 Mathematical and Statistical Methods group (Biometris), Wageningen University and Research, Wageningen, The Netherlands; 2 Department of Data Science and Biostatistics, Julius Centre, UMC Utrecht, Utrecht, The Netherlands; 3 Department of Mathematics, Radboud University Nijmegen, Nijmegen, The Netherlands

**Keywords:** Metabolites, Network reconstruction, Repeated measures

## Abstract

****Introduction**:**

The metabolome is an intermediate between DNA variation and clinical phenotypes. Metabolomics have been widely used in biomedical studies for reflecting physiological changes in response to variation coming from various sources, such as diet, environment, time, and lifestyle. While lifestyle factors contribute a considerable part of the metabolic variation, current human studies lack information estimating lifestyle, mainly because it is not strictly defined.

****Objective**:**

In this work, metabolite concentrations are measured at two time points (2007 and 2014). Additionally, SNP data together with self-reports on dietary behavior. By having measurements over time, as well as all main sources of metabolic variation (diet, genetics), both time-effects and lifestyle-effects can be estimated. Since lifestyle and time effects can be estimated under this setting, we are interested in identifying metabolites sharing similar relationships to diet and lifestyle, using network analysis.

****Methods**:**

The correlation between repeated measurements is modeled using a random intercepts linear mixed model, with dietary preferences, genetics, and time as fixed effects. The random intercepts can be defined as the lifestyle, and represent the part of the metabolic variation which is not due to diet, genetics, and time and is subject-specific. The part of every metabolite relevant to diet and lifestyle instead of the original values is used as input values to network estimation methods.

****Conclusions**:**

This work demonstrates how correcting for several sources of metabolic variation, allows us to look for residual variation and build networks with meaningful metabolite groups sharing similar association to diet and lifestyle.

## Introduction

The metabolome is the complete collection of metabolites, which are intermediates or end products of metabolic pathways associated with cells, tissues or organs (Nielsen and Jewett, 2007). The metabolome captures information from all functional levels of a cell (Nielsen, 2003). It has been used as a tool for biomarker detection, drug discovery and safety, diet strategies and genetic disease testing (Tebani et al., 2016) because it reflects the underlying biochemical activity. Since it dynamically interacts with other molecules and the environment (Beisken et al., 2015), it occupies a unique place in systems biology, where an organism is viewed as a complex web of interacting molecular entities (Nielsen and Jewett, 2007). Additionally, metabolites themselves, by being sensitive to variation coming from genetics, time, and environmental stimuli, are widely used for assessing any type of systematic change in biochemical activity (Tebani et al., 2016). The variability induced by these sources of variation, produces fluctuations in the metabolite concentrations that spread through enzymatic reactions and create correlation patterns (Morgenthal et al., 2006). Metabolic network analysis tools that recover these correlation patterns have been described in the literature by representing metabolites as nodes in a graph and their relationships as edges connecting the nodes (Morgenthal et al., 2006; Ursem et al., 2008; Weng et al., 2019; Watson et al., 2013; Floegel et al., 2014; Bartzis et al., 2017).

### Objective and motivation

In this study, our interest is in recovering meaningful metabolite patterns using network analysis, while metabolite measurements are taken repeatedly on the same individuals over time. Previously, we incorporated information on the study design when metabolite networks were estimated (Bartzis et al., 2017). Extending this approach, here we work with repeated metabolite measurements. In this setting, metabolite concentrations of a subject are dependent (due to time) and this dependence should be taken into account when the data are analyzed. We use a linear mixed effects model for the metabolite concentrations, allowing us to estimate time-specific and subject-specific random effects. When time, diet, and genetics are included in the model, the subject-specific effects represent all remaining unmeasured shared sources of metabolic variation, i.e., lifestyle. Lifestyle can be defined as the collection of smaller environmental effects, like physical activity, sleeping patterns, interests, etc. Since it is thought of in an abstract way, it is usually not quantifiable and common approaches ignore it as a part of random variation.

Here, by having a repeated measures design together with information on diet and genetics, time and lifestyle effects can be estimated. By working in the network framework, our interest is in recovering meaningful metabolite patterns associated with specific dietary preferences and lifestyle. Additionally, working with the estimated time effects allows the recovery of metabolite sets that change similarly over time.

This work is motivated by the DILGOM (DIetary, Lifestyle, and Genetic determinants of Obesity and Metabolic syndrome) study. More information can be found in Inouye et al. (2010) and Kettunen et al. (2012). This Finnish population study investigates how nutrition, diet, lifestyle, psychosocial factors, environment, and genetics are linked to obesity and the metabolic syndrome.

### Contribution

By using dietary preferences we have a better understanding of the extent to which the metabolic patterns are influenced by diet (Pallister et al., 2015). To date, several studies have assessed the interplay between diet and metabolism in the context of nutritional epidemiology (Guertin et al., 2014; Floegel et al., 2014; Pallister et al., 2015; Schmidt et al., 2015; Xu et al., 2010). A standard technique for quantifying dietary patterns is by making use of Food Frequency Questionnaires (FFQ) with Exploratory Factor Analysis (EFA) (Hu, 2002; Newby and Tucker, 2004). By exploring correlation patterns among food items, common underlying dietary factors are identified and a score summarizing dietary patterns is typically determined (Hu, 2002). Here, we use these scores for summarizing dietary information and studying the interplay between metabolites regarding dietary and lifestyle choices, as well as the interrelationship among the metabolites with regard to time.

Despite the extended use of network analysis in modeling metabolic pathways, to our knowledge, only a few other studies have investigated the link between diet and serum metabolites using network analysis (Watson et al., 2013; Floegel et al., 2014; Wang et al., 2018; Weng et al., 2019). This allows the determination of how habitual factors associate to metabolite classes (lipoproteins, amino acids, etc). In this work, we use lifestyle information and take into account the genetic contribution to metabolite concentrations. While studying the metabolome using network analysis, we address genetic variation by using Polygenic Risk Scores (PRS) (Dudbridge, 2013) for summarizing genetic information.

For network estimation, we use undirected networks, where the relationship between two connected nodes is symmetric. The estimation of metabolite connection patterns in this paper is based on the graphical LASSO (glasso) (Hastie et al., 2009; Friedman et al., 2008). Compared to methods that are based on the observed correlation structure (and therefore recovering edges based on indirect associations), glasso is based on partial correlation and recovers edges while avoiding spurious associations.

### Overview

The rest of the paper is organized as follows. In Sect. 2 the motivating dataset is described. In Sect. 3, we propose an extension to the method of Bartzis et al. (2017) in the repeated measures setting for selecting information relevant to certain sources prior to network estimation. Additionally, we review existing methods for summarizing genetic and environmental variation. In Sect. 4, we demonstrate how to select specific variation parts in metabolite data, which deviates from standard approaches in nutritional epidemiology by addressing simultaneously metabolite variation induced by time, genetics, lifestyle, and diet. We conclude the article with a discussion in Sect. 5.

## Data

In this section, we will describe the data used in this study coming from an epidemiological cohort, namely DILGOM, a subset of the FINRISK study (Inouye et al., 2010; Kettunen et al., 2012). In this study, metabolite data measured at two time points were available (2007 and 2014) together with information on food preferences (described by a Food Frequency Questionnaire; FFQ), genetic information (single nucleotide polymorphisms; SNPs), as well as age and biological gender (hereafter indicated as sex). Since only two time points (2007 and 2014) are included in our data, time was considered a binary factor. Similarly, sex was also a binary factor. Finally, we use the genetic information (SNP information) to calculate a genetic quantitative score per subject to capture the different genetic background of individuals. Our interest here is in studying metabolite patterns with regard to dietary and lifestyle choices. As part of the data cleaning process, we excluded subjects who were diagnosed with diabetes and had outlying fasting glucose levels (over 10mmol/l). In addition only subjects with complete information on age, sex and food preferences were further considered. After applying the exclusion criteria, 364 subjects (171 males and 193 females) aged between 25 and 74 years (median 51) at the first time point (2007) and 211 subjects (104 males and 107 females) aged between 32.11 and 81.23 years (median 59.1) at the second time point (2014) were considered for further analysis. For each time point (2007 and 2014), the continuous age values were transformed into a binary factor (one for individuals younger than 50 years old and two for individuals equal to or older than 50 years old). We opted for the age of 50 due to its alignment with (i) the recommended age for certain health screenings and check-ups for monitoring age-related health issues (Chen et al., 2019), and (ii) the average age of onset of menopause among women (Bromberger et al., 1997).

### Food frequency questionnaire

A FFQ was given to the participants of the study in 2007 to record their eating and drinking patterns. The FFQ contained the eating frequency of 40 food items (e.g. pizza, meat, chocolate, etc.) in a scale from one (rarely) to eight (more than four times per day) and the daily drinking frequency of 15 beverages under a typical serving (e.g. cups of coffee, glasses of milk, etc.). The FFQ will be subjected to Exploratory Factor Analysis (EFA) to extract factors that correspond to interpretable diets.

### SNP data

For computing the individual genetic effect on the subject-specific metabolite profiles, information on approximately 38 million genotyped and imputed SNPs was available for every individual. Since the metabolite variation explained by each SNP separately is rather small, PRS is used as described in Sect. 3.2.1.

### Metabolite data

Metabolite data in both time points were measured by nuclear magnetic resonance and comprise absolute quantitative measurements on 228 serum metabolites (groups of lipoproteins, lipids, amino-acids, fatty acids and others). Metabolites that were expressed as percentages (78 metabolites) were removed and their concentration levels were retained. Additionally, we removed 83 lipid particle subfractions (due to high correlation) and only the total lipid concentrations per particle size were used. Furthermore, five more metabolites were removed since they were expressed either as fractions or they were highly correlated with retained metabolites. Finally, seven metabolites were eliminated for not having information on any possible association with any SNP (Kettunen et al., 2012); hence the data that were considered for analysis consisted of 55 metabolites.

## Methods

Often, one might be interested in metabolite variation from a specific source, such as, for example, diet (Bartzis et al., 2017). By estimating networks based on this source of variation, metabolites that associate to that source of variation in the same way, will share an edge. For estimating networks that contain information on parts relevant to this source of variation, we take two steps: 1) we identify an appropriate model for the responses (metabolites here) and 2) we select the part of the responses that we are interested in to extract a network. A network consists of a set of *p* nodes (metabolites) connected by a set of edges (relationships between metabolites) and is represented by a symmetric $p\times p$ matrix ${\textbf {A}}$ (adjacency matrix) of ones and zeroes depending on whether the corresponding metabolites are connected. In addition, an intensity matrix ${\textbf {W}}$ can be considered where the elements represent the intensity of the connection between the nodes (essentially a weighted version of ${\textbf {A}}$). In this paper, we consider as intensity the stability of the estimated edges (i.e., the probability of an edge being true, as calculated by the network estimation method in 3.3). The number of neighbors of a node *i* which is the sum of row or column *i* of matrix $\textbf{A}$, is called degree. By taking into account both the degree and the intensity of the edges, the strength of node *i* ($s_i$) can be calculated as the sum of row or column *i* of matrix $\textbf{W}$. Following the estimation of the intensity and adjacency matrices, groups of closely interconnected metabolites are usually identified using a clustering algorithm where the similarity measure is based on $\textbf{W}$ (as described in Sect. 3.3.1).

### Estimating subject-specific metabolite effects

At the first step, since we work in the repeated measures framework, the correlation between the measurements is modeled using linear mixed effect models with random intercepts, representing the shared unobserved factors.

Let $\textbf{Y}^{(p)}$ be the vector of concentrations of the *p*th metabolite over two timepoints. Further, assume that $\textbf{T}$ is the covariable denoting the discrete point in time where the metabolite concentrations were measured for each individual. Finally, we can have *m* other covariables, e.g. genetics, dietary preferences, age, and sex.

For the *p*th metabolite we model the within subject correlation by using subject-specific effects from a random-intercepts linear mixed model. For identifying the part of the metabolite concentrations associated to dietary and lifestyle choices we then fit the following model:

1 $$\begin{aligned} \textbf{Y}^{(p)}=\beta _{0}^{(p)}+&\beta _{1}^{(p)}\textbf{Age}+\beta _{2}^{(p)}\textbf{Sex}+\beta _{3}^{(p)}\textbf{T}+\beta _{4}^{(p)}\textbf{F} +\nonumber \\&\beta _{5}^{(p)}\textbf{G}+\beta _{6}^{(p)}\textbf{Age}\circ \textbf{Sex}+\beta _{7}^{(p)}\textbf{Age}\circ \textbf{T}+\nonumber \\&\beta _{8}^{(p)}\textbf{Age}\circ \textbf{F}+\beta _{9}^{(p)}\textbf{Age}\circ \textbf{G}+\beta _{10}^{(p)}\textbf{Sex}\circ \textbf{T}+\nonumber \\&\beta _{11}^{(p)}\textbf{Sex}\circ \textbf{F}+\beta _{12}^{(p)}\textbf{Sex}\circ \textbf{G}+\beta _{13}^{(p)}\textbf{T}\circ \textbf{F}+\nonumber \\&\beta _{14}^{(p)}\textbf{T}\circ \textbf{G}+\beta _{15}^{(p)}\textbf{F}\circ \textbf{G}+\textbf{u}^{(p)}+\varvec{\varepsilon }^{(p)}, \end{aligned}$$

where $\varvec{\varepsilon }^{(p)} \sim \mathcal{N}\big(0, \sigma^2_{(p)}\big)$ is the random noise, and $\circ$ is the Hadamard product. In model 1, $\textbf{u}^{(p)} \sim \mathcal{N}\big(0, \sigma^2_{u(p)}\big)$ are the subject-specific effects representing all unmeasured shared factors. $\textbf{G}$, represents the genetics, and $\textbf{F}$ the dietary patterns. $\textbf{Age}$ and $\textbf{Sex}$ are the vectors containing the age and sex of the subjects, respectively. Note that the subject-specific effects ($\textbf{u}^{(p)}$) are conditioned on multiple terms, i.e., age, sex, time, genetics, diet, and their interactions. Therefore, this source of metabolic variation is not associated with them, thus is a variable accounting for individual metabolic differences, hence lifestyle. In principle, lifestyle is hard to estimate since it depends on many factors that are not available to us. Here, by having measurements over time we were able to estimate it as the random intercepts of the linear mixed effects model conditioned on all other sources of variation. Finally, the time interval between the repeated measures is the same for all subjects.        

The relevant metabolite part related only to dietary and lifestyle choices in the linear mixed model 1 that will be used for estimating metabolite networks is given by:

2 $$\begin{aligned} \tilde{\textbf{Y}}_L^{(p)}= & \hat{\beta }_{4}^{(p)}\textbf{F}+\hat{\beta }_{8}^{(p)}\textbf{Age}\circ \textbf{F}+\hat{\beta }_{11}^{(p)}\textbf{Sex}\circ \textbf{F}+\nonumber \\ &\hat{\beta }_{13}^{(p)}\textbf{T}\circ \textbf{F} +\hat{\beta }_{15}^{(p)}\textbf{F}\circ \textbf{G}+\hat{\textbf{u}}^{(p)}. \end{aligned}$$

The quantification of the dietary (F) and genetic (G) parts for inclusion in model 1, using EFA and PRS, is described in Sect. 3.2.

### Identifying diets with exploratory factor analysis

Different diet patterns strongly influence disease risks and have an effect on health. Many studies have examined the associations between intakes of individual foods (Hu et al., 1999) and health or lifestyle. However the intake of one food or nutrient is often correlated with the intake of another (Randall et al., 1992; Hu et al., 1999). Therefore, dietary patterns can be identified by using the correlation among the foods, typically by using EFA (Slattery et al., 1998; Hu et al., 1999, 2000; Williams et al., 2000).

EFA is a latent variable model attempting to explain complex relationships between observed variables by using an unobserved structure (Rencher, 2003). The dimension of the latent vector is lower than the dimension of the observable variables. In EFA, we have a set of observed variables (e.g. food preferences) generated by a number of unobserved latent variables (e.g. diets). The idea is to identify and summarize the unobserved variables that explain the dependence between the observed variables.

For *p* observed variables, and *m* unobserved factors, let $\textbf{o}$ be the observed centered eating frequencies, i.e., $\textbf{o}=(\textbf{o}_1,\textbf{o}_2,\ldots,\textbf{o}_p)^\top$. For notational simplicity, we leave out the notation for observations. Also let $\textbf{f}=(\textbf{f}_1,\textbf{f}_2,\ldots,\textbf{f}_m)^\top$ be the unobserved diets. Factor analysis is expressing each food frequency as a linear combination of the diets, i.e.,

3 $$\begin{aligned} \textbf{o} = \textbf{L}\textbf{f}+\varvec{\epsilon }, \end{aligned}$$

where $\textbf{L}$ is the $p\times m$ loadings matrix measuring the dependence of observed variables on factors, $\varvec{\epsilon }=(\varvec{\epsilon }_1,\varvec{\epsilon }_2,\ldots,\varvec{\epsilon }_p)^\top$ is the random error distributed as $\mathcal {N}(\textbf{0},\mathbf {\Psi })$ with $\mathbf {\Psi }$ being diagonal, $\textbf{f} \sim \mathcal {N}(\textbf{0},\mathbf {\Phi })$, and $\textrm{Cor}(\textbf{f}, \varvec{\epsilon }) = \textbf{0}$. Factor analysis is expressing the covariance of the observed variables ($\textrm{Cov}(\textbf{o})=\mathbf {\Sigma }$) in terms of $\textbf{L}$, $\mathbf {\Phi }$ and $\mathbf {\Psi }$, i.e., $\mathbf {\Sigma }=\mathbf {L\Phi L}^\top +\mathbf {\Psi }$. The number of latent factors can be chosen based on the factor interpretability. In order to do so, we need to carefully examine the loading matrix for different selections of number of factors in combination with usage of the scree plot (Cattell, 1966). A typical practice for making the results more interpretable is by employing factor rotation, where the estimated loading matrix is generally transformed by multiplying it by an orthogonal or non orthogonal matrix. Factor analysis was conducted using the principal axis factoring (PA) method, followed by “oblimin" rotation to allow for correlated factors. The analysis was performed using the *psych* package for R (Revelle, 2021).

The latent diets $\textbf{F}$ can be quantified by using the “ten Berge" factor scores (Ten Berge et al., 1999). In that way, the correlation of the dietary patterns is preserved when the sample factor score correlations are computed.

#### Polygenic risk scores (PRS)

A popular practice for uncovering the genetic variants that influence metabolite concentrations are the metabolite-based genome-wide association studies, i.e., mGWAS (Raffler et al., 2015). It has been shown that in GWAS, common single nucleotide polymorphisms (SNPs) exhibit significant roles in determining phenotypic variation (Chatterjee et al., 2016). Although separate SNPs typically explain only a moderate proportion, a combination of them can explain a substantial part of the phenotypic variation (Chatterjee et al., 2016; Dudbridge, 2013). Therefore, polygenic risk scores (PRS) are widely used for summarizing genetic effects $\textbf{G}$ from a set of markers associated to a phenotype of interest.

Typically, for estimating a PRS (denoted as $\textbf{G}$ here) for a phenotype $\textbf{Y}$ (e.g. the concentration levels of a metabolite), we have a set of *l* SNPs ($\textbf{S}=(\textbf{S}_{1},\ldots,\textbf{S}_{l})^{\top }\in \{0,1,2\}^{l}$). For each of the markers, the effect size ($\mathbf {\eta }$) is determined, e.g. by the estimated coefficients from a linear regression of $\textbf{Y}$ on each of the *l* SNPs.

For computing the PRS ($\textbf{G}$), a subset of the top $\tilde{l}$ associated SNPs is used (Euesden et al., 2015). Then, the linear combination of the top SNPs weighted by their corresponding effect sizes is calculated. For subject *i* the PRS is computed as:

4 $$\begin{aligned} \textbf{G}_{i}=\sum ^{\tilde{l}}_{j=1} \mathbf {\eta }_{j}\textbf{S}_{ij}. \end{aligned}$$

Note that $\textbf{G}$ contains genetic information related to $\textbf{Y}$ and can be used for further analysis.

### Network estimation using glasso

In this paper, for network estimation we use glasso (Hastie et al., 2009; Friedman et al., 2008), which is based on partial-correlations using conditional independence and recovers conditional associations between the nodes.

In glasso, it is assumed that the metabolite concentrations follow a multivariate Gaussian distribution with mean vector $\varvec{\mu }$ and variance-covariance matrix $\mathbf {\Sigma }$. The network is estimated as the non-zero entries of the precision matrix ($\mathbf {\Theta }=\mathbf {\Sigma }^{-1}$). For estimating the precision matrix, typically a penalized log-likelihood approach is used which produces a sparse estimate of $\mathbf{\Theta}$. The penalized version of the log-likelihood that is maximized (Hastie et al., 2009; Friedman et al., 2008) uses a LASSO penalty as follows:

5 $$\begin{aligned} \ell _\lambda (\varvec{\Theta })\propto \log|\varvec{\Theta }|-\text {tr}(\varvec{K}\varvec{\Theta })-\lambda||\varvec{\Theta }||_1, \end{aligned}$$

where $\textbf{K}$ is the sample covariance matrix of $\tilde{\textbf{Y}}_L$ and $\lambda$ is a non-negative tuning parameter controlling the sparsity of the estimated precision matrix $\hat{\mathbf {\Theta }}$. Here we use the stability approach for regularization selection (StARS) (Liu et al., 2010) for obtaining the regularization parameter making the network sparse and replicable under random sampling. In StARS multiple overlapping subsamples of the data are selected and sparse networks are estimated for $\lambda$ values in a grid. For the optimal $\lambda$, the stability of an edge can be calculated as the observed relative frequency of it being estimated over the subsamples.

#### Module identification

A network usually consists of a set of modules that have closely interconnected metabolites. A typical way of identifying them is by using the two-step dynamic hybrid algorithm (Langfelder et al., 2008) on the metabolite dendrogram resulting by using $\textbf{W}$ as the similarity matrix. An alternative way for module identification is by using the Girvan–Newman algorithm (Newman & Girvan, 2004) based on edge-betweenness implemented in the rags2ridges R-package (Peeters et al., 2022). Using different module identification algorithms allows us to get a better understanding of the network’s modular structure and possibly identify sets of nodes that consistently emerge. In this study, we base $\textbf{W}$ on StARS. We first recover the adjacency matrix ${\textbf {A}}$ for the estimated optimal $\lambda$ resulting to a stable network under random subsampling. The symmetric ${\textbf {A}}$ matrix contains 1s and 0s depending on whether the corresponding nodes are connected for the optimal $\lambda$ value. The non-zero entries (edges) are then weighted by the relative estimated frequency of the edge being present over all subsamples used. Subsequently, the matrix containing the relative estimated frequency of the stable estimated edges is $\textbf{W}$ and can be used for module identification.

### Network characterization

We now consider three measures to describe a network or a module, namely density, centralization and heterogeneity (Dong and Horvath, 2007). For a square symmetric $p\times p$ matrix $\textbf{M}$, let *s* be the strength of a node (row sum of $\textbf{W}$) and $\bar{s}$*s*. The quantities in Table 1 are then computed for network or module characterization. For density, a value close to one indicates high interconnectedness between the nodes in the network/module. High values in Centralization, denote a star shaped network, i.e., the network contains one highly connected node. Finally, the heterogeneity indicates the amount of edge diversity in the network/module.

**Table 1:** Measures that can be used for describing a network or part of a network

Quantity	Formula	High values denote that..	Range
Density	$\sum _{i}\sum _{j}\dfrac{M_{ij}}{p(p-1)}$	$\textbf{M}$ is highly interconnected	$\left[ -1,1\right]$
Centralization	$\dfrac{1}{p}\left( \text{ max }(s)-\bar{s} \right)$	$\textbf{M}$ contains hub node(s)	$\left[ -1,1\right]$
Heterogeneity	$\dfrac{\sqrt{\textsf {var}(s)}}{\bar{s}}$	the values in $\textbf{M}$ are diverse	$\left[ 0, \infty \right)$

## Application to data

In this section, we will use the methods of Sect. 3 for analyzing, visualizing, and evaluating the conditional correlation structure of the metabolite data subject to: i) dietary and lifestyle variation, ii) time variation, while addressing for individuals genetic background. Prior to that, we need to estimate dietary ($\textbf{F}$) and genetic information ($\textbf{G}$) for adding them together with age, sex, and time in model 1, while lifestyle is estimated by the empirical Bayes estimates of the random intercepts of this model.

**Fig. 1:**
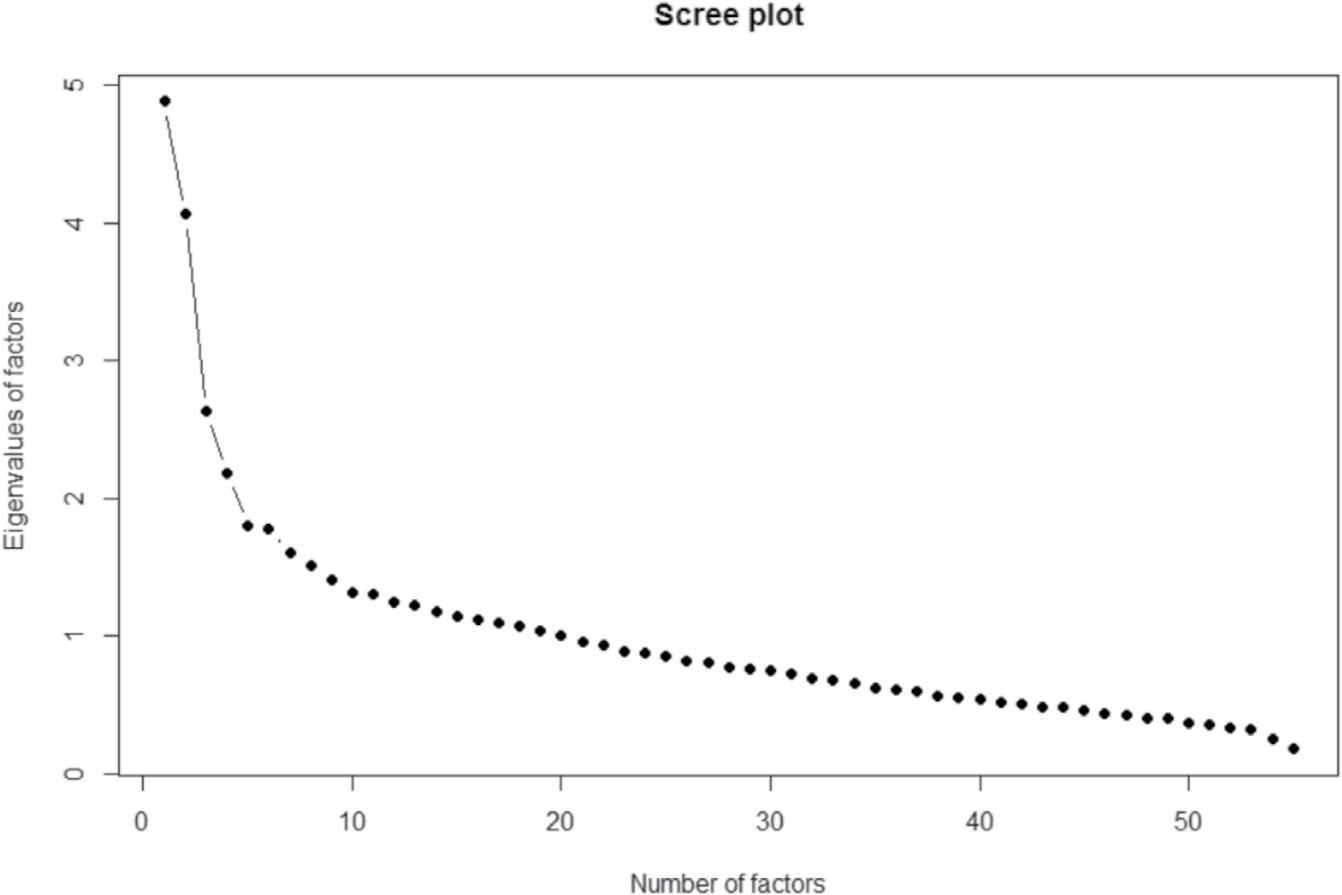
Scree plot for selecting number of dietary profiles

The FFQ data were used for identifying latent dietary patterns emerging from the complex relationships between the 55 observed eating/drinking items. To estimate dietary information we used EFA. The loading matrix $\textbf{L}$ was estimated and can be seen in Table 2. The visual inspection of the scree plot (Fig. 1) revealed that the number of possible latent diets that could be recovered from the data were five to six. Here, we chose six diets. For the rest of the paper we refer to these six diets as: $\text {F}_1$, $\text {F}_2$, $\text {F}_3$, $\text {F}_4$, $\text {F}_5$, $\text {F}_6$.

The dietary patterns were determined after closely examining the factor loadings: a) fast food (F1), b) vegetarian (F2), c) high caloric (F3), d) fish (F4), e) juice (F5), and f) balanced (F6) respectively. Finally, we assumed that individuals do not change dietary patterns in a 7-year period time. That means the dietary scores are the same in the two time points.

For estimating the individuals genetic background, we first selected 48 SNPs based on a mGWAS study (Kettunen et al., 2012) on 8,330 Finnish individuals (DILGOM was part of the study) and Linkage Disequilibrium pruning for dealing with correlation between SNPs. In Eq. 4, the PRS ($\textbf{G}_i$) was computed for every available metabolite with $\mathbf {\eta }$ obtained from the supplementary material of Kettunen et al. (2012).

### Metabolite networks for separate timepoints

**Fig. 2:**
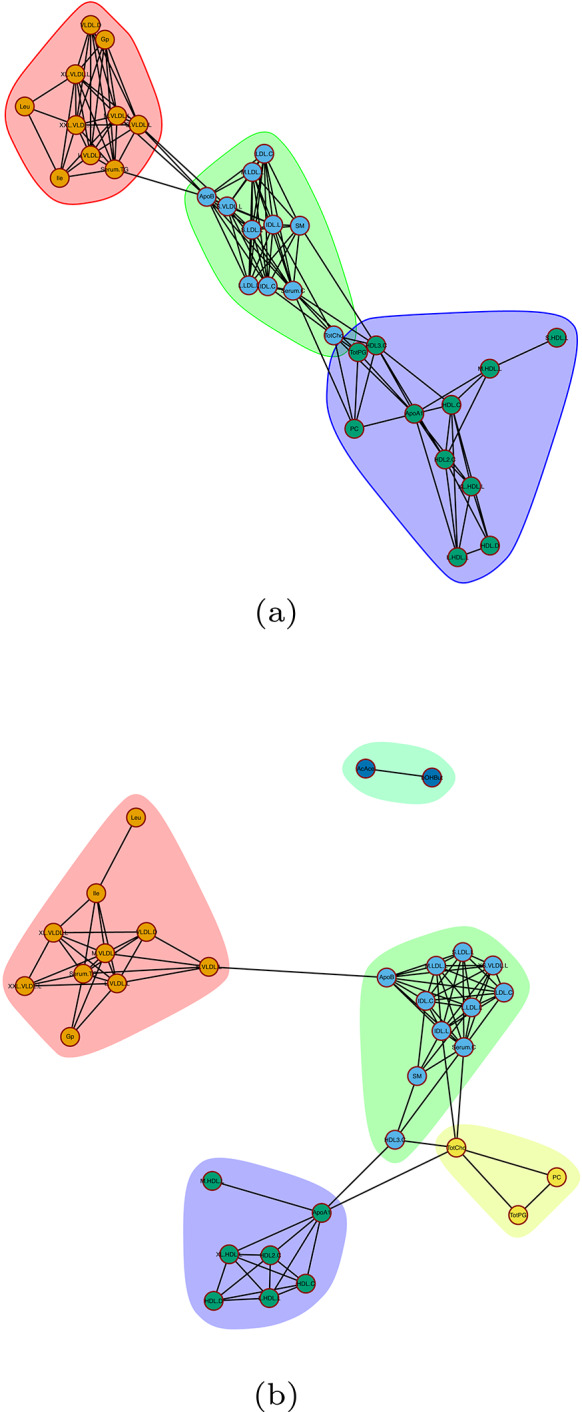
Estimated metabolite networks and cluster identification using the Girvan–Newman algorithm when age and sex have been accounted for. The metabolite networks concern different time points **a** 2007, **b** 2014

In order to analyze the data, we first estimated metabolite networks with regard to the different time points, i.e., 2007 and 2014. The estimated metabolite networks can be seen in Fig. 2. For those networks, the metabolites were corrected for age and sex differences by keeping the residuals of a linear model with each metabolite as response and Age, Sex, and their interaction as predictor variables.

In the case of the network concerning the metabolite measurements in 2007, three modules have been identified using the Girvan–Newman algorithm. The first mainly consisted of VLDLs, the second of Lipoproteins, while the last one had mainly high density lipoproteins. For the metabolite network when using the 2014 measurements, four modules were identified using the Girvan–Newman algorithm. Those modules had high overlap with the ones from the 2007 network.

As the data concern metabolite measurements of the same individuals over different time points, the intra-subject correlation is not utilized when networks are estimated separately. Additionally, repeated measures allow us to estimate time effects and study interactions between time, genetics, and dietary patterns. By utilizing a detailed mixed model which suitably models the repeated measure data, random intercepts are estimated and can be interpreted as the residual metabolic variation not attributed to dietary, demographic or genetic information, i.e., lifestyle. The estimated lifestyle information can further be used together with dietary information to estimate metabolite networks subject to those two sources of metabolic variation.

### Metabolite networks subject to dietary patterns and lifestyle

For estimating networks of metabolites with respect to dietary and lifestyle information, model 1 was first fitted on the data. Then, for the *p*th metabolite, the values $\tilde{\textbf{Y}}_L^{(p)}$ (Model 2) were used for network estimation. To estimate a metabolite network using glasso, we first selected the tuning parameter $\lambda$ controlling the network sparsity in glasso ($\lambda = 0.533$).

**Fig. 3:**
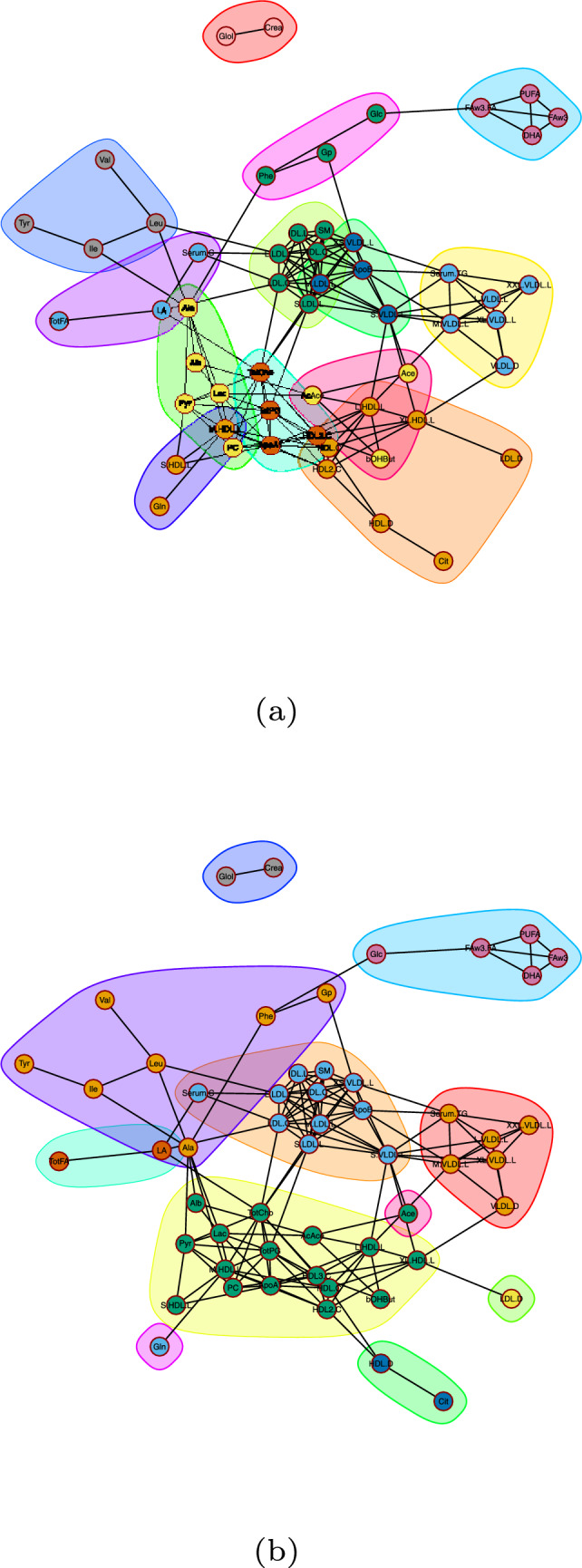
Estimated metabolite networks with respect to dietary and lifestyle variation when cluster identification is performed using the **a** two-step dynamic hybrid algorithm **b** Girvan–Newman algorithm

Using the two-step dynamic hybrid algorithm, 12 modules were identified (VLDL1, VLDL2, lipid metabolism, lipoproteins, $\omega$-3 FA, carbohydrate metabolism, glycogenesis, AA, ketone bodies, BCAAs, HDL; Fig. 3a). In Table 3, the clusters of interconnected metabolites were characterized by using our descriptive measures (density, centralization and heterogeneity) for clusters that contain five or more metabolites. The complete network displays a small value in terms of density (0.10) and high value for heterogeneity (0.64) compared to the identified modules (high densities and low heterogeneity). This implies a good module separation (high density within modules compared to low for nodes in different modules).

Using the Girvan–Newman algorithm, eight modules were identified (VLDL, lipoproteins, $\omega$-3 FA, HDL/ketone bodies/lipid metabolism, AA; Fig. 3b). In Table 4, the clusters of interconnected metabolites were again characterized by density, centralization and heterogeneity. As in the two-step dynamic hybrid algorithm, here the complete network displays again a small value in terms of density (0.10) and high value in heterogeneity (0.64). Contrarily to the networks for the separate time points, the identified modules had high densities and low heterogeneity pointing again to good module separation.

### Comparison between networks for separate time points and networks subject to dietary patterns and lifestyle

Compared to the case of separate networks per time point, here we estimate different parts of metabolic variation before we reconstruct metabolic networks. This resulted in better separated networks, i.e., higher number of modules. The estimated modules can be better identified as the different metabolic classes, i.e., amino acids, VLDLs, HDLs, $\omega$-3 fatty acids, etc.

**Fig. 4:**
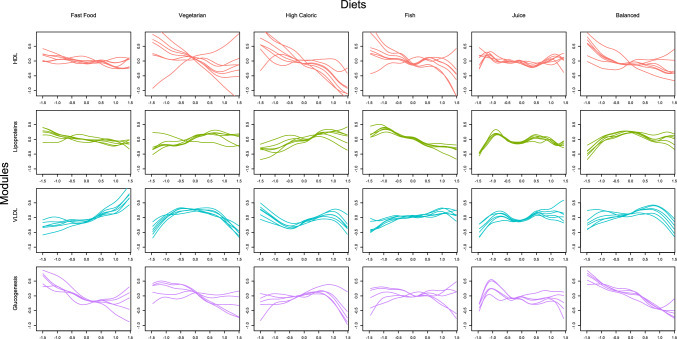
Metabolite profile plots when the metabolite clusters have been identified using the dynamic cut tree algorithm. The relationship of the concentration levels of every metabolite (*y*-axis) to each diet (factor scores on the *x*-axis) is depicted using a spline. All metabolites belonging in the same module have the similar relationships to the diets

For the estimated modules using the two-step hybrid algorithm, the HDL module (Cit, HDL.C, HDL.D, HDL2.C, L.HDL.L, LDL.D, XL.HDL.L), the lipoproteins module (IDL.C, IDL.L, L.LDL.L, LDL.C, S.LDL.L, SM), and one of the VLDL modules (L.VLDL.L, M.VLDL.L, Serum.TG, VLDL.D, XL.VLDL.L, XXL.VLDL.L) had the highest amount of metabolites (7, 6, and 6 respectively). By inspecting the metabolite profiles (Fig. 4), it can be observed that the metabolic profiles for the HDL module were not as homogeneous as for the other modules (density was estimated at 0.46). Conversely the profiles for the lipoproteins and VLDL modules were much more homogeneous, seen also by their density (0.96 and 0.86, respectively).

Interestingly, using the Girvan–Newman algorithm for module identification, the lipid metabolism module was clustered together with the ketone bodies and the high-density lipoproteins resulting in 16 metabolites within the cluster. The lipoproteins module in this case contained 11 metabolites and had again high density (0.76).

**Fig. 5:**
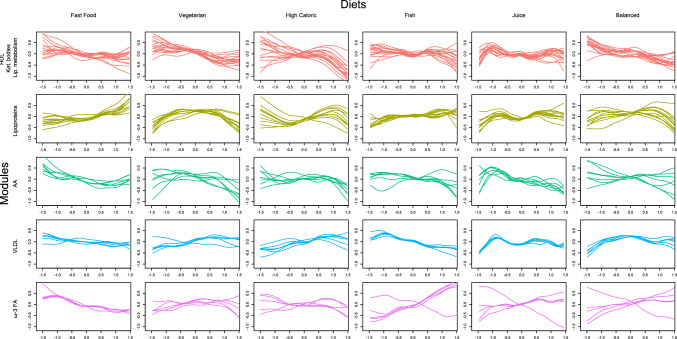
Metabolite profile plots when the metabolite clusters have been identified using the Girvan–Newman algorithm. The relationship of the concentration levels of every metabolite (*y*-axis) to each diet (factor scores on the *x*-axis) is depicted using a spline. All metabolites belonging in the same module have the similar relationships to the diets

The VLDL and HDL modules appeared to have on average opposite associations to every diet. The negative association might stem from HDL transporting very-low-density lipoprotein to the liver, where they are broken down. Mainly, by following a fast-food, a vegetarian, or a high-caloric diet, a negative association to HDL was observed in our data (Figs. 4 and 5).

## Discussion

In this work, our interest was on recovering metabolite networks under a repeated measures setting. By having information on various sources of variation (age, sex, time, genetic background, dietary preferences), lifestyle was able to be estimated as the random effects of a linear mixed effects model with the metabolite concentrations as response variable. By estimating time effects and quantifying lifestyle, metabolite networks were estimated with regard to lifestyle and dietary preferences while addressing for individual’s genetic differences. For network estimation, we considered the glasso method which is based on conditional independence. The network estimation method was applied to human metabolite data and interconnected modules having the same relationship to diets and lifestyle were identified using two methods: i) the two-step dynamic hybrid algorithm, and ii) the Girvan–Newman algorithm. Obtained networks and modules were described in terms of density, centralization, and heterogeneity.

As the data were collected at two time-points (2007 and 2014), we estimated metabolite networks for the separate time-points, as a benchmark, for comparing to the more elaborate model which utilizes information on time, genetics, and dietary patterns. We observed that the separate networks had fewer, more dense modules. Contrarily, a network accounting for different sources of variation resulted in more modules that were also more homogenous in terms of constituent metabolites. When information related to dietary preferences and lifestyle was retained in metabolite networks, several groups of biologically associated metabolites were clustered together.

By working on the repeated measures setting, networks subject to time variation can also be estimated. Identified modules will contain metabolites that change similarly over time. In order to perform such analysis, the metabolite part related to time variation that can be used for network estimation is given by:

6 $$\begin{aligned} \tilde{\textbf{Y}}_T^{(p)}= & \hat{\beta }_{3}^{(p)}\textbf{T}+\hat{\beta }_{7}^{(p)}\textbf{Age}\circ \textbf{T}+\hat{\beta }_{10}^{(p)}\textbf{Sex}\circ \textbf{T}+\hat{\beta }_{13}^{(p)}\textbf{T}\circ \textbf{F} +\nonumber \\ &\hat{\beta }_{14}^{(p)}\textbf{T}\circ \textbf{G}. \end{aligned}$$

Although, several studies have examined the interplay between diet and metabolism, to our knowledge, this is the first attempt studying metabolite patterns in the network framework while simultaneously modeling diet ($\textbf{F}$), lifestyle ($\textbf{u}$), and genetics ($\textbf{G}$), when concentrations are measured over time. Under this design, metabolite measurements are dependent and this dependence should be taken into account when the data are analyzed. By using linear mixed effects models, we were able to decompose and select the part of metabolic variation relevant to specific covariables, i.e., $\textbf{F}$ and $\textbf{u}$. Established relationships were identified and metabolites were separated by their different biochemical classes.

However, some limitation should be noted. First, we assumed the individual dietary scores, as estimated by the EFA, to be the same between the two time points. Overall dietary patterns tend to be relatively stable over time, while it is suggested that in repeated measures studies, dietary information is to be reevaluated after at least seven years (Weismayer et al. 2006). Second the FFQ contains self-reported data, which may have limitations such as recall bias and social desirability bias. Despite the limitations, FFQs are still valuable tools for assessing dietary intake in large-scale epidemiological studies.

Zooming into variation of specific covariables allows us to interpret and infer different metabolite aspects. Using this framework while working on the metabolome, more can be done. For example, by identifying clusters of metabolites responding similarly to specific dietary patterns, network analysis can help detect diet-responsive biomarkers. This can be valuable for evaluating metabolic health and/or risk for chronic conditions. Additionally, in metabolite identification and characterization, when an unidentified metabolite is included in the network, its properties regarding different aspects can be deduced by carefully examining the edges connected to the metabolite, with respect to different variation sources. Working in the same framework for the reconstruction of metabolite networks in humans and plants (Bartzis et al., 2017), in the future we plan to use information from the graphical structure of lower leveled omic sources (gene or marker level) besides accounting for the study design. This will allow us to use an extra level of information for reconstructing metabolite networks.

**Table 2:** Loadings matrix

Food item	Fast Food (F1)	Vegetarian (F2)	High Caloric (F3)	Fish (F4)	Juice (F5)	Balanced (F6)
Chocolate	**0**.**60**					
Other candies	**0**.**55**					
Sweet biscuits	**0**.**52**	**0**.**29**				
Other sweet pastry	**0**.**45**					
Salty snacks	**0**.**44**	$-$**0.30**				
Ice cream puddings	**0**.**43**					
Cereals or muesli	**0**.**37**		−**0.29**			
Store bought ready meal	**0**.**37**					
Pizza	**0**.**32**	−**0.28**				
Flavoured yoghurt	**0**.**31**					
Yeast bread.graham bread						
Cola light/day						
Chocolate milk/day						
Fruits		**0**.**55**				
Fresh or frozen berries		**0**.**53**				
Porridge		**0**.**49**				
Sweet coffee bread or pies	**0**.**31**	**0**.**43**				
Low fat cheese		**0**.**36**				**0**.**28**
Burgers	**0**.**29**	−**0.34**				
Rye bread & rye crisp		0.31				
Non-flavoured yoghurt		**0**.**31**				
Cooked vegetables		**0**.**30**				
Sour milk/day		**0**.**28**				
Vegetarian food		**0**.**26**				
Energy drink/day						
Sausages			** 0.62**			
Cutlets			**0**.**59**			
Meat			**0**.**43**			**0**.**33**
Cooked or smashed potatoes		**0**.**40**	**0**.**42**			
Roasted potatoes or french fries			**0**.**42**			
Salty pies and pastry	**0**.**30**		**0**.**36**			
White bread			**0**.**29**			
Eggs			**0**.**29**			
Coffee/day			**0**.**26**			
Milk/day						
Other cheese						
Coffee/day			**0**.**26**			
Tap water/day						
Soft drink/day						
Low alcohol /day						
Fish and other fish food combined				**0**.**91**		
Salmon & rainbow trout				**0**.**64**		
Other fish				**0**.**58**		
Herring				**0**.**48**		
Tea/day						
Bottled water/day						
Fruit and berry juices					**0**.**76**	
Poultry meat						**0**.**60**
Cold cuts			**0**.**30**			**0**.**44**
Pasta or rice	**0**.**26**					**0**.**36**
Fresh salad. Fresh vegetables		**0**.**29**				**0**.**32**
Salad dressing or oil						
Low calorie soft drink/day						
Well water/day						

Measures the dependence of observed variables on factors. Loadings with absolute value above 0.25 have been indicated

**Table 3:** Characterization of the modules when the Girvan–Newman algorithm is used

Module	Density	Centralization	Heterogeneity	Nr of nodes
HDL ^a^	0.46	0.18	0.50	7
Lipoproteins^b^	0.96	0.03	0.04	6
VLDL2^c^	0.86	0.12	0.19	6
gluconeogenesis ^d^	0.63	0.28	0.37	5
Complete Network	0.10	0.11	0.64	54

Only modules with five or more metabolites have been reported

^a^ Cit, HDL.C, HDL.D, HDL2.C, L.HDL.L, LDL.D, XL.HDL.L

^b^ IDL.C, IDL.L, L.LDL.L, LDL.C, S.LDL.L, SM

^c^ L.VLDL.L, M.VLDL.L, Serum.TG, VLDL.D, XL.VLDL.L, XXL.VLDL.L

^d^ Ala, Alb, Lac, PC, Pyr

**Table 4:** Characterization of networks and modules subject to dietary patterns and lifestyle, when the Girvan–Newman algorithm is used

Module	Density	Centralization	Heterogeneity	Nr of nodes
HDL/Ket. bodies/Lip. metabolism ^a^	0.37	0.23	0.43	16
Lipoproteins^b^	0.76	0.20	0.29	11
AA ^c^	0.30	0.16	0.48	7
VLDL ^d^	0.86	0.12	0.19	6
$\omega$-3 FA ^e^	0.67	0.23	0.39	5
Complete Network	0.10	0.11	0.64	54

Only modules with five or more metabolites have been reported

^a^ AcAce, Alb, ApoA1, bOHBut, HDL.C, HDL2.C, HDL3.C, L.HDL.L, Lac, M.HDL.L, PC, Pyr, S.HDL.L, TotCho, TotPG, XL.HDL.L

^b^ ApoB, IDL.C, IDL.L, L.LDL.L, LDL.C, M.LDL.L, S.LDL.L, S.VLDL.L, Serum.C, SM, XS.VLDL.L

^c^ Ala, Gp, Ile, Leu, Phe, Tyr, Val

^d^ L.VLDL.L, M.VLDL.L, Serum.TG, VLDL.D, XL.VLDL.L, XXL.VLDL.L

^e^ DHA, FAw3, FAw3.FA, Glc, PUFA

## Data Availability

The DILGOM data are included in the THL Biobank (https://www.thl.fi/en/web/thl-biobank). The data used in the present study can be made available upon request.

## References

[CR1] Nielsen, J., & Jewett, M.C. (2007). The role of metabolomics in systems biology, 1–10.

[CR2] Nielsen, J. (2003). It is all about metabolic fluxes. *Journal of Bacteriology,**185*(24), 7031–7035. 14645261 10.1128/JB.185.24.7031-7035.2003 PMC296266

[CR3] Tebani, A., Abily-Donval, L., Afonso, C., Marret, S., & Bekri, S. (2016). Clinical metabolomics: The new metabolic window for inborn errors of metabolism investigations in the post-genomic era. *International Journal of Molecular Sciences,**17*(7), 1167. 27447622 10.3390/ijms17071167 PMC4964538

[CR4] Beisken, S., Eiden, M., & Salek, R. M. (2015). Getting the right answers: Understanding metabolomics challenges. *Expert Review of Molecular Diagnostics,**15*(1), 97–109. 25354566 10.1586/14737159.2015.974562

[CR5] Morgenthal, K., Weckwerth, W., & Steuer, R. (2006). Metabolomic networks in plants: Transitions from pattern recognition to biological interpretation. *Biosystems,**83*(2), 108–117. 16303239 10.1016/j.biosystems.2005.05.017

[CR6] Ursem, R., Tikunov, Y., Bovy, A., Van Berloo, R., & Van Eeuwijk, F. (2008). A correlation network approach to metabolic data analysis for tomato fruits. *Euphytica,**161*(1–2), 181–193.

[CR7] Weng, Y. J., Gan, H. Y., Li, X., Huang, Y., Li, Z. C., Deng, H. M., Chen, S. Z., Zhou, Y., Wang, L. S., Han, Y. P., Tan, Y. F., Song, Y. J., Du, Z. M., Liu, Y. Y., Wang, Y., Qin, N., Bai, Y., Yang, R. F., Bi, Y. J., & Zhi, F. C. (2019). Correlation of diet, microbiota and metabolite networks in inflammatory bowel disease. *Journal of Digestive Diseases,**9*, 447–459. 10.1111/1751-2980.12795 10.1111/1751-2980.12795 31240835

[CR8] Watson, E., MacNeil, L. T., Arda, H. E., Zhu, L. J., & Walhout, A. J. M. (2013). Integration of metabolic and gene regulatory networks modulates the c. elegans dietary response. *Cell,**153*(1), 253–266. 10.1016/j.cell.2013.02.050 23540702 10.1016/j.cell.2013.02.050 PMC3817025

[CR9] Floegel, A., Wientzek, A., Bachlechner, U., Jacobs, S., Drogan, D., Prehn, C., Adamski, J., Krumsiek, J., Schulze, M., & Pischon, T. (2014). Linking diet, physical activity, cardiorespiratory fitness and obesity to serum metabolite networks: Findings from a population-based study. *International Journal of Obesity,**38*(11), 1388–1396. 24608922 10.1038/ijo.2014.39 PMC4229626

[CR10] Bartzis, G., Deelen, J., Maia, J., Ligterink, W., Hilhorst, H. W., Houwing-Duistermaat, J.-J., van Eeuwijk, F., & Uh, H.-W. (2017). Estimation of metabolite networks with regard to a specific covariable: Applications to plant and human data. *Metabolomics,**13*(11), 129. 28989335 10.1007/s11306-017-1263-2 PMC5610247

[CR11] Inouye, M., Kettunen, J., Soininen, P., Silander, K., Ripatti, S., Kumpula, L. S., Hämäläinen, E., Jousilahti, P., Kangas, A. J., & Männistö, S. (2010). Metabonomic, transcriptomic, and genomic variation of a population cohort. *Molecular Systems Biology,**6*(1), 441. 21179014 10.1038/msb.2010.93 PMC3018170

[CR12] Kettunen, J., Tukiainen, T., Sarin, A.-P., Ortega-Alonso, A., Tikkanen, E., Lyytikäinen, L.-P., Kangas, A. J., Soininen, P., Würtz, P., & Silander, K. (2012). Genome-wide association study identifies multiple loci influencing human serum metabolite levels. *Nature Genetics,**44*(3), 269–276. 22286219 10.1038/ng.1073 PMC3605033

[CR13] Pallister, T., Sharafi, M., Lachance, G., Pirastu, N., Mohney, R. P., MacGregor, A., Feskens, E. J., Duffy, V., Spector, T. D., & Menni, C. (2015). Food preference patterns in a UK twin cohort. *Twin Research and Human Genetics,**18*(06), 793–805. 26412323 10.1017/thg.2015.69

[CR14] Guertin, K.A., Moore, S.C., Sampson, J.N., Huang, W.-Y., Xiao, Q., Stolzenberg-Solomon, R.Z., Sinha, R., & Cross, A.J. (2014). Metabolomics in nutritional epidemiology: Identifying metabolites associated with diet and quantifying their potential to uncover diet-disease relations in populations. The American Journal of Clinical Nutrition, 078758. 10.3945/ajcn.113.078758 PMC4144099 24740205

[CR15] Schmidt, J. A., Rinaldi, S., Ferrari, P., Carayol, M., Achaintre, D., Scalbert, A., Cross, A. J., Gunter, M. J., Fensom, G. K., & Appleby, P. N. (2015). Metabolic profiles of male meat eaters, fish eaters, vegetarians, and vegans from the epic-oxford cohort. *The American Journal of Clinical Nutrition,**102*(6), 1518–1526. 26511225 10.3945/ajcn.115.111989 PMC4658459

[CR16] Xu, J., Yang, S., Cai, S., Dong, J., Li, X., & Chen, Z. (2010). Identification of biochemical changes in lactovegetarian urine using 1h NMR spectroscopy and pattern recognition. *Analytical and Bioanalytical Chemistry,**396*(4), 1451–1463. 20016880 10.1007/s00216-009-3338-z

[CR17] Hu, F. B. (2002). Dietary pattern analysis: A new direction in nutritional epidemiology. *Current Opinion in Lipidology,**13*(1), 3–9. 11790957 10.1097/00041433-200202000-00002

[CR18] Newby, P., & Tucker, K. L. (2004). Empirically derived eating patterns using factor or cluster analysis: A review. *Nutrition Reviews,**62*(5), 177–203. 15212319 10.1301/nr.2004.may.177-203

[CR19] ...Wang, D. D., Zheng, Y., Toledo, E., Razquin, C., Ruiz-Canela, M., Guasch-Ferré, M., Yu, E., Corella, D., Gómez-Gracia, E., Fiol, M., Estruch, R., Ros, E., Lapetra, J., Fito, M., Aros, F., Serra-Majem, L., Clish, C. B., Salas-Salvadó, J., Liang, L.,… Hu, F. B. (2018). Lipid metabolic networks, Mediterranean diet and cardiovascular disease in the PREDIMED trial. *International Journal of Epidemiology,**47*, 1830–1845. 10.1093/ije/dyy198 30428039 10.1093/ije/dyy198 PMC6280948

[CR20] Dudbridge, F. (2013). Power and predictive accuracy of polygenic risk scores. *PLoS Genet,**9*(3), 1003348. 10.1371/journal.pgen.1003348 PMC3605113 23555274

[CR21] Hastie, T., Tibshirani, R., & Friedman, J. H. (2009). *The elements of statistical learning: data mining, inference, and prediction* (Vol. 2). Springer.

[CR22] Friedman, J., Hastie, T., & Tibshirani, R. (2008). Sparse inverse covariance estimation with the graphical lasso. *Biostatistics,**9*(3), 432–441. 18079126 10.1093/biostatistics/kxm045 PMC3019769

[CR23] Chen, C., Stock, C., Hoffmeister, M., & Brenner, H. (2019). Optimal age for screening colonoscopy: A modeling study. *Gastrointestinal Endoscopy,**89*(5), 1017–1025. 30639539 10.1016/j.gie.2018.12.021

[CR24] Bromberger, J. T., Matthews, K. A., Kuller, L. H., Wing, R. R., Meilahn, E. N., & Plantinga, P. (1997). Prospective study of the determinants of age at menopause. *American Journal of Epidemiology,**145*(2), 124–133. 9006309 10.1093/oxfordjournals.aje.a009083

[CR25] Kettunen, J., Tukiainen, T., Sarin, A.-P., Ortega-Alonso, A., Tikkanen, E., Lyytikäinen, L.-P., Kangas, A. J., Soininen, P., Würtz, P., & Silander, K. (2012). Genome-wide association study identifies multiple loci influencing human serum metabolite levels. *Nature Genetics,**44*(3), 269–276. 22286219 10.1038/ng.1073 PMC3605033

[CR26] Hu, F. B., Rimm, E., Smith-Warner, S. A., Feskanich, D., Stampfer, M. J., Ascherio, A., Sampson, L., & Willett, W. C. (1999). Reproducibility and validity of dietary patterns assessed with a food-frequency questionnaire. *The American Journal of Clinical Nutrition,**69*(2), 243–249. 9989687 10.1093/ajcn/69.2.243

[CR27] Randall, E., Marshall, J.R., Brasure, J., & Graham, S. (1992). Dietary patterns and colon cancer in western New York. 10.1080/01635589209514227 1296200

[CR28] Slattery, M. L., Boucher, K. M., Caan, B. J., Potter, J. D., & Ma, K.-N. (1998). Eating patterns and risk of colon cancer. *American Journal of Epidemiology,**148*(1), 4–16. 9663397 10.1093/aje/148.1.4-a

[CR29] Hu, F. B., Rimm, E. B., Stampfer, M. J., Ascherio, A., Spiegelman, D., & Willett, W. C. (2000). Prospective study of major dietary patterns and risk of coronary heart disease in men. *The American Journal of Clinical Nutrition,**72*(4), 912–921. 11010931 10.1093/ajcn/72.4.912

[CR30] Williams, D. E., Prevost, A. T., Whichelow, M. J., Cox, B. D., Day, N. E., & Wareham, N. J. (2000). A cross-sectional study of dietary patterns with glucose intolerance and other features of the metabolic syndrome. *British Journal of Nutrition,**83*(03), 257–266. 10884714 10.1017/s0007114500000337

[CR31] Rencher, A.C. (2003). Methods of multivariate analysis **492**.

[CR32] Cattell, R. B. (1966). The scree test for the number of factors. *Multivariate Behavioral Research,**1*(2), 245–276. 26828106 10.1207/s15327906mbr0102_10

[CR33] Revelle, W. (2021). Psych: Procedures for Psychological, Psychometric, and Personality Research. Northwestern University, Evanston, Illinois. Northwestern University. R package version 2.1.9. https://CRAN.R-project.org/package=psych.

[CR34] Ten Berge, J. M., Krijnen, W. P., Wansbeek, T., & Shapiro, A. (1999). Some new results on correlation-preserving factor scores prediction methods. *Linear Algebra and its Applications,**289*(1–3), 311–318.

[CR35] Raffler, J., Friedrich, N., Arnold, M., Kacprowski, T., Rueedi, R., Altmaier, E., Bergmann, S., Budde, K., Gieger, C., & Homuth, G. (2015). Genome-wide association study with targeted and non-targeted NMR metabolomics identifies 15 novel loci of urinary human metabolic individuality. *PLoS Genet,**11*(9), 1005487. 10.1371/journal.pgen.1005487 PMC4564198 26352407

[CR36] Chatterjee, N., Shi, J., & García-Closas, M. (2016). Developing and evaluating polygenic risk prediction models for stratified disease prevention. *Nature Reviews Genetics,**17*(7), 392–406. 27140283 10.1038/nrg.2016.27 PMC6021129

[CR37] Euesden, J., Lewis, C. M., & O’reilly, P. F. (2015). Prsice: Polygenic risk score software. *Bioinformatics,**31*(9), 1466–1468. 25550326 10.1093/bioinformatics/btu848 PMC4410663

[CR38] Liu, H., Roeder, K., Wasserman, L. (2010). Stability approach to regularization selection (stars) for high dimensional graphical models. In: Advances in Neural Information Processing Systems, pp. 1432–1440. PMC4138724 25152607

[CR39] Langfelder, P., Zhang, B., & Horvath, S. (2008). Defining clusters from a hierarchical cluster tree: the dynamic tree cut package for R. *Bioinformatics,**24*(5), 719–720. 18024473 10.1093/bioinformatics/btm563

[CR40] Newman, M. E., & Girvan, M. (2004). Finding and evaluating community structure in networks. *Physical Review E,**69*(2), Article 026113. 10.1103/PhysRevE.69.026113 14995526

[CR41] Peeters, C. F. W., Bilgrau, A. E., & van Wieringen, W. N. (2022). rags2ridges: A one-stop-l2-shop for graphical modeling of high-dimensional precision matrices. *Journal of Statistical Software,**102*, 1–32.

[CR42] Dong, J., & Horvath, S. (2007). Understanding network concepts in modules. *BMC Systems Biology,**1*(1), 24. 17547772 10.1186/1752-0509-1-24 PMC3238286

[CR43] Weismayer, C., Anderson, J. G., & Wolk, A. (2006). Changes in the stability of dietary patterns in a study of middle-aged Swedish women. *The Journal of Nutrition,**136*(6), 1582–1587. 16702325 10.1093/jn/136.6.1582

